# Alexithymia and Its Associations With Depression, Suicidality, and Aggression: An Overview of the Literature

**DOI:** 10.3389/fpsyt.2019.00203

**Published:** 2019-04-11

**Authors:** Laura Hemming, Gillian Haddock, Jennifer Shaw, Daniel Pratt

**Affiliations:** ^1^Division of Psychology and Mental Health, School of Health Sciences, Faculty of Biology, Medicine and Health, University of Manchester, Manchester Academic Health Science Centre, Manchester, United Kingdom; ^2^Greater Manchester Mental Health NHS Foundation Trust, Manchester Academic Health Science Centre, Manchester, United Kingdom

**Keywords:** alexithymia, depression, suicide, aggression, emotion dysregulation

## Abstract

Depression affects around 4–10% of the general population in England. Depression can often lead to behaviors and thoughts related to suicide and aggression, which have a social and economic burden to the United Kingdom. One construct that has been theorized as having an association with these behaviors is alexithymia. People with alexithymia have difficulties identifying and describing their emotional experiences. To date, there is no consensus on types or causes of alexithymia. Whilst the literature evidences a strong relationship between alexithymia and suicidality and aggression, little is known about the nature of this relationship. The present article will attempt to describe the extant literature on this relationship, drawing out some of the contentions and unanswered questions.

## Introduction

It is estimated that around 4–10% of the population in England will experience depression at some point in their lives (1). Risk factors for depression include experiencing stressful life events, particular personality types, family history of depression, giving birth, loneliness, alcohol and drugs, and physical illness (2–8). Depression, along with other mental health problems, are thought to constitute the largest single source of world economic burden, with an estimated global cost of £1.6 trillion (9).

A related, but separate national issue is that of self-harm, including death from suicide. It has been reported that depression serves as a risk factor for these behaviors, with the most common primary diagnoses of patients who self-harm (72%) and die by suicide (45%) being affective disorders such as bipolar disorder and depression (10, 11). Self-harm is thought to be a particular issue for children and young people, with a reported 41,218 self-harm episodes in 10-24 year olds in 2017–2018 (Hospital Episode Statistics)^1^. In adults, suicide is the most common cause of death for men aged 20–49 years in England and Wales (12) and it is estimated that one person in 15 has made a suicide attempt at some point in their life (1). Self-harm is thought to have several adverse outcomes both for the individual and for those around them. For example, people who engage in self-harm are often thought to be at risk of a range of outcomes including repetitive self-harm (13) and death by suicide (14). Furthermore, experiencing a suicide in the family is thought to incite significant disruption to social relationships (15). Specifically, those bereaved by suicide have been found to be more likely to attempt suicide themselves (16), and also less likely to be in contact with mental health services (17). Whilst there has been no attempt to quantify the costs associated with self-harm, it has been estimated that service users who self-harmed had an extra £3,524 spent on them over 6 months than service users who did not (18). Moreover, it is estimated that the direct and indirect costs of each suicide costs £1.67 million in England (19).

Other risky behaviors, such as aggression and violence, pose several problems for society. For instance, there were 1.3 million reported violent crimes in England and Wales in 2015 (12). It has been estimated that the economic impact of violent crime is as much as $14.3 trillion globally which equates to 12.6% of world GDP (20). The cost to the families of victims in the UK is estimated at around £1.3 million (21). Several theories have been suggested to explain the risk factors for violence, which highlight the roles of individual as well as environmental factors (22).

It has been observed that suicidality and aggression are intrinsically linked with one another. For instance, within clinical samples, it has frequently been found that there is considerable overlap in patients who have experienced suicidality and patients who have experienced aggression (23–25). This led Plutchik et al. (26) to develop the two-stage model of suicide and violence, which proposes that both suicide and aggression represent the expression of the same underlying aggressive impulse. It is the absence or presence of specific correlates that determines which activity the individual engages in. Although some support for such models has been demonstrated [e.g., Gvion and Apter (27)], this has been inconsistent, highlighting the need to examine more specific aspects of emotional experience and their contributions toward suicide and violence behaviors.

One factor which has been posited as having an important role in the pathway to suicidality and aggression is alexithymia. Alexithymia is a concept which stemmed from the field of psychosomatics and can broadly be defined as the inability to understand and describe one's feelings (28). It is difficult to estimate the prevalence of alexithymia due to there being no clear diagnostic criteria, however various studies have estimated the prevalence at between 10 and 19% (29–32).

This brief report will summarize the literature relating to alexithymia. Specifically, it will aim to address some of the nuances of alexithymia, including the different types of alexithymia posited, and also review some of the research which has examined the relationship between alexithymia and depression, suicide, and violence. This report is not intended to comprise a comprehensive systematic review but is instead intended to provide a commentary on the literature to date pertaining to alexithymia and depression, suicide and violence.

## What is Alexithymia?

### Definition and Origins

Translated, the term “alexithymia” literally means a lack (a) of words (lexi) for emotions (thymia). Whilst the concept is similar to that of emotion dysregulation, it is generally accepted that emotion dysregulation refers to a more broad range of behaviors, whereas alexithymia is a more nuanced and specific type of emotion dysregulation (33). Since its inception, alexithymia has been defined as comprising five main features (34); (i) a difficulty in identifying one's emotions (ii) a difficulty in describing self-feelings verbally (iii) a reduction or incapability to experience emotions (iv) an externally orientated cognitive style, and (v) poor capacity for fantasizing or symbolic thought.

Alexithymia was originally rooted in the field of Psychosomatics with MacLean (35) being one of the first to suggest a link between a person's emotional experiences and their physical bodily complaints. He recognized that a large proportion of his psychosomatic patients reported a limited ability to use verbal or symbolic cues to discuss and identify their emotions. Sifneos (36) described how, during his work in a psychiatric clinic in the 1960's and 70's, he was struck by psychosomatic patients “marked difficulty in finding appropriate words to describe how they felt, giving the impression that they did not understand the meaning of the word “feeling”” (pg. 137). Sifneos goes on to detail how he termed these experiences “alexithymia”. This led to further research which explored the link between these experiences and psychosomatic complaints.

Following Sifneos's original observations, there have been a number of empirical attempts at quantifying the relationship between alexithymia and somatisation. A review of such literature found a small to moderate relationship between the two phenomena with correlation coefficients ranging from −0.26 to 0.60 (37).

### Types of Alexithymia

It is thought that there are two distinct types of alexithymia: primary (or trait) alexithymia and secondary (or state) alexithymia. Freyberger (38) was the first to demarcate these types, stating that primary alexithymia should be viewed as a dispositional factor whilst secondary alexithymia should be viewed as a defense mechanism which is open to manipulation within treatment.

To date, the majority of research has approached alexithymia as a stable personality trait. From this viewpoint, alexithymia is thought to be developmental in nature, and emerges during childhood or early adult years (38). Several authors claim to have illustrated this continuous aspect of alexithymia by evidencing both relative and absolute stability of the trait. For instance, Salminen et al. (39) administered a rating scale for alexithymia, the Toronto Alexithymia Scale (TAS-20) (40) to a group of general psychiatric outpatients at the time of admission and again 1 year later. Whilst during this time there was a significant decrease in psychological distress, they found there was no significant change in alexithymia scores. Similar results have also been found in general population samples (41) and a range of specific groups including irritable bowel syndrome (IBS) patients (42), patients with depressive disorder (43) and in students (44).

In contrast, research on secondary alexithymia posits that alexithymia does not arise during development but can emerge at any time in life as a consequence of distressing life events and/or trauma. Some studies have confirmed this, suggesting that alexithymia may develop in response to overwhelming stress in order to avoid experiencing unbearable emotion (45, 46). Several researchers have used longitudinal studies to disprove alexithymia as a stable personality trait. For instance alexithymia scores have been found to decrease in association with improvement in clinical symptomatology in follow-up periods across a range of clinical groups, such as substance use disorder (47), anxiety disorders (48), and various other mental disorders (48). Thus, this body of literature suggests that alexithymia may develop during times of psychological distress but later subside concomitantly with psychological and somatic symptomatology.

## What is Alexithymia Associated With?

### Depression

Depression and alexithymia are often described as similar constructs, with some authors even going as far as to suggest that subscales of alexithymia do no more than measure the pre-defined concept of depression (49).

For example, Parker et al. (50) found small to moderate correlations (*r* = 0.28 for student sample and *r* = 0.47 for psychiatric outpatient sample) between alexithymia and depression, however the results of their factor analysis showed that alexithymia was a construct separate and distinct from the construct of depression. More recently, Li et al. (51) conducted a meta-analysis of studies using both clinical and general population samples and found that alexithymia scores were moderately correlated to scores for depression severity (*r* = 0.46). In summary, the research to date suggests that alexithymia and depression are distinct, but closely related constructs (30, 41, 50, 52, 53).

One question still remains, however, as to whether this relationship demonstrates the vulnerability hypothesis (that alexithymia predisposes people to depression) the reactivity hypothesis (that depression causes the onset of alexithymia), or whether alexithymia and depression simply co-exist with one another (41). The majority of studies conclude that their evidence supports the vulnerability hypothesis suggesting that alexithymia should be seen as primary. For instance, Gilanifar and Delavar (53) found that women who were experiencing alexithymia had a 2.6 times greater risk of experiencing depression and Günther et al. (54) found in their prospective study that a high alexithymia score at baseline was a significant predictor of depression at follow-up. Despite this, the majority of studies examining this relationship have failed to use a prospective design and can therefore not conclude definitively on the causality of the relationship. Furthermore, a number of studies have found that alexithymia has been lower at follow-up than at baseline, suggesting that alexithymia levels decrease with associated reduction of symptoms of depression (41, 43, 55). This could instead suggest that alexithymia is secondary in nature and occurs as a reaction to psychopathology, in this case, depression.

### Suicidality

Several studies have shown that alexithymia may be associated with suicidality in general population samples (56–58) and in a variety of clinical samples, including those with; obsessive-compulsive disorder, post-traumatic stress disorder, depression, adjustment disorder, binge eating disorder, schizophrenia, traumatic brain injury, and substance abuse (59–67).

Studies have largely focussed on either the relationship between alexithymia and suicide ideation or on the relationship between alexithymia and suicide behavior, with some studies examining both. This evidence supports the notion that alexithymia is more strongly related to suicide ideation than suicide behavior, with correlation coefficients reported to range from 0.28 to 0.75 (65, 67–71) for ideation. Despite this, a small number of studies have also found a relationship between alexithymia and suicide behavior, namely with self-harm or suicide deaths, with correlation coefficients ranging from 0.09 to 0.22 (72–74).

To date, there is little research which investigates the underlying mechanisms which link these two phenomena. For instance, what leads the person with alexithymia to consider suicide as a solution to their distress? As already discussed, there is a strong body of research which suggests that alexithymia and depression are very closely related constructs (30). Several studies have therefore found that severity of depression mediates the link between alexithymia and suicide (56, 68, 72, 75, 76). However, more recent studies have found that the link between alexithymia and suicide remains, even after accounting for depression (67).

There are also several other personal and situational factors which may impact on the relationship between alexithymia and suicide. For instance, low self-esteem has been frequently found to be associated separately with both alexithymia (77–79) and suicidality (80, 81). Lower levels of social support have also been found to be separately associated with alexithymia (82, 83), and suicide (84, 85). Finally, there is evidence to suggest that attachment style is related to both alexithymia (86, 87) and suicide (88, 89).

There is an evident need to map the pathway between alexithymia and suicide including the interplay with several other variables of interest.

### Aggression

Research investigating the relationship between alexithymia and aggression has been attenuated by variation in the definitions used for violence. For instance, some studies have approached aggression as a stable trait in the individual whilst others have approached it as a momentary state response. This difference underpins the various methodologies that have been utilized to assess the relationship between alexithymia and violence.

Some studies have examined the prevalence of alexithymia in populations assumed to be inherently aggressive, including; adolescents with severe disruptive behavior (90), male delinquent adolescents (91), adolescent sexual offenders (92), perpetrators of cyber-bullying (93), and adult offenders (94, 95). Each of these populations was found to have a higher prevalence of alexithymia compared to a healthy control group, with Cohen's D effect sizes ranging from 0.25 to 0.84, suggesting an association between alexithymia and aggression. Despite this, a large proportion of these studies were conducted with adolescents, and Moriarty et al. (92) suggested that it may be that alexithymia is simply more prevalent in adolescents in general, as opposed to violent adolescents.

Other studies have included a measure of aggression and used this to investigate the quantitative relationship between alexithymia and aggression, rather than compare the prevalence of alexithymia between assumed aggressive and non-aggressive (control) samples. These studies showed a relationship between alexithymia and aggression in the following populations; violent forensic outpatients, *r* = 0.23 (96), people experiencing substance dependence, *r* = 0.42–0.47 (97, 98), males with antisocial personality disorder, *r* = 0.22 (99) and veterans with traumatic symptoms, *r* = 0.32 (100). More specifically, these studies found that it was primarily the difficulties with identifying feelings aspect of alexithymia that was related to aggression. Conversely, these studies found little evidence for an association between aggression and the domain of difficulties with externally orientated thinking, which, if experienced, is related to a restricted imagination and paucity of fantasy.

Several other factors may have an impact on the relationship between alexithymia and aggression. For instance, it has been found that emotion dysregulation may mediate the relationship between self-esteem and aggression (101). More specifically, low self-esteem has been found to be associated separately with both alexithymia and aggression (102, 103). Higher levels of social support may serve to inhibit the relationship between alexithymia and aggression, as higher levels of social support have been found to protect against the development of violence (104). Finally there is evidence to suggest that attachment style may impact on the relationship between alexithymia and aggression, with an anxious attachment style directly impacting on antisocial behavior (105, 106).

## Treatment of Alexithymia

It has been frequently observed that people experiencing alexithymia may find it difficult to engage with and benefit from psychological therapy (28, 107–109) and that alexithymia may also inhibit the therapeutic effect of prescribed medication (107, 110, 111). Despite this, recent evidence suggests that levels of alexithymia did not significantly differ between patients who chose medication, psychotherapy or declined treatment (112). Future research should aim to explore this area in more detail, with the view to evidence exactly how alexithymia may (or may not) attenuate the impact of various treatments.

Furthermore, due to alexithymia being a trans-diagnostic construct, few studies have investigated interventions with a primary focus upon alexithymia, and instead have evaluated the secondary impact of treatments for various psychological disorders. Such research has found that alexithymia scores have reduced following treatment for bulimia nervosa (113), panic disorder (114), obsessive compulsive disorder (115) and depression (116). However, if alexithymia is to be viewed as secondary in nature, then these decreases in scores may be a result of the reduction of psychopathological symptoms, rather than the therapy having a direct effect on alexithymia itself.

Despite this, a small body of research has emerged which is primarily focussed on targeting alexithymia during psychotherapy. Parker et al. (117) suggested that emotional intelligence may be a protective factor in the relationship between alexithymia and various clinical diagnoses. This theory is supported by findings that the teaching of emotional components to increase emotional intelligence led to a significant reduction in alexithymia scores (118). Further to this, research has attempted to address which elements of psychological therapy may be most useful for patients with alexithymia, for instance advocating that therapy be group based (119, 120), and adopt supportive and educational approaches as opposed to interpretive approaches (38, 121, 122).

## Conclusion

It is clear that clinically, there is still much to be learnt about alexithymia and its relationship with a range of related phenomena. Firstly, is alexithymia a continuous and stable trait independent of psychological or somatic symptomology that is developed during childhood? Or is it instead a reactive state induced by trauma and distress at any age, which serves to defend against intense and upsetting emotions? This impacts on treatment options. For example, should we be focussing on early childhood interventions which target the child's emotional environment and parenting to encourage emotional expression? Or should we instead be treating alexithymia after it has occurred—perhaps through teaching elements of emotional intelligence following the experience of a traumatic event?

Given this inconclusive evidence on the etiology of alexithymia, it is important for future research to explore this issue in greater detail. Such research would benefit from a longitudinal prospective design which aims to test the stability of alexithymia over a long period of time, and also measure concomitant psychopathology. Analysis should aim to investigate the interplay between psychopathology symptoms and alexithymia symptoms.

Second, we need to learn more about how alexithymia relates to other constructs. Alexithymia is thought to be closely and intrinsically related to depression, suicide and aggression. The relationships between these phenomena also have implications for treatment. For instance, it is important to establish the theoretical and conceptual differences between alexithymia and depression in order to tailor treatment to the correct construct. Moreover, it is important to establish whether the relationship between alexithymia and suicide is predominantly explained by concomitant depression and if not what are the appropriate treatment options? Furthermore, are the building blocks of the relationship between alexithymia and suicidality/aggression the same, or do they differ?

Finally, there is a need for a wealth of research to be conducted into the treatment of alexithymia. Interventions should aim to be informed by future research, some of which has been proposed in this review, to ensure that treatments target alexithymia specific features, and not those of similar but separate constructs such as depression or emotion dysregulation. Once such an intervention is developed, it is important to evaluate its efficacy in thorough detail in a range of populations with differing clinical diagnoses. This is important to ensure that the intervention is targeting the trans-diagnostic phenomena of alexithymia, and not just diagnosis specific symptoms.

The current review does not claim to be comprehensive and is intended only as a narrative description of some of the contentions within the field of literature examining alexithymia. Beyond this, it is apparent that a comprehensive systematic review is needed to synthesize all the extant data available and to provide support for some of the theories posited in this review. In addition to this, a strong programme of investigative research is required to clarify the relationship between alexithymia and suicidality and aggression.

## Author Contributions

LH wrote the first draft of the manuscript. GH, JS, and DP wrote sections of the manuscript. All authors contributed to manuscript revision, read and approved the submitted version.

### Conflict of Interest Statement

The authors declare that the research was conducted in the absence of any commercial or financial relationships that could be construed as a potential conflict of interest.

## Footnotes

^1^https://fingertips.phe.org.uk/search/self%20harm#page/11/gid/1/pat/6/par/E12000005/ati/102/are/E08000025.
